# Evaluation of an Isolation Program of Hepatitis C Virus Infected Hemodialysis Patients in Some Hemodialysis Centers in Egypt

**DOI:** 10.5402/2013/395467

**Published:** 2012-10-31

**Authors:** Amin R. Soliman, Mohamed Momtaz Abd Elaziz, Mona I. El lawindi

**Affiliations:** ^1^Department of Medicine, Cairo Hospital, 41 Manial Street, Cairo 11451, Egypt; ^2^Department of Public Health, Cairo Hospital, 41 Manial Street, Cairo 11451, Egypt

## Abstract

*Introduction*. Hepatitis C virus (HCV) infection is a significant cause of morbidity and mortality in hemodialysis (HD) patients. Several studies demonstrated nosocomial transmission of HCV among HD patients. *Aim*. We aimed to evaluate the isolation program of HCV seropositive patients among a group of Egyptian haemodialysis patients to decrease the incidence of HCV seroconversion. *Methods*. One hundred and fourteen HCV seronegative patients who were receiving regular haemodialysis in different four haemodialysis units in Egypt. The first group included forty six patients on regular hemodialysis in two centers following strict isolation of the HCV seropositive patients, and the second group included sixty eight patients on regular hemodialysis in the other two centers not following this strict isolation. All these patients were followed up over a period of 36 months. *Results*. There was a significantly higher incidence of HCV seroconversion of patients on hemodialysis in units not following strict isolation of HCV seropositive patients (42.9%) than those on regular hemodialysis in units following strict isolation (14.8%). *Conclusions*. In HD units with a high prevalence of HCV+ patients, strict isolation of HCV+ patients in combination with implementation of universal prevention measures can limit the spread of HCV infection in HD patients.

## 1. Introduction

Hepatitis C virus infection has been reported to be the most common blood born pathogen all over the world [1]. In Egypt, infection with HCV has become the most important public health problem nowadays with the overall prevalence of anti-HCV in Egypt in 1993 was 13.6% [2] Haemodialysis patients are at a high risk of infection by many blood borne pathogens. Some studies on haemodialysis patients in the United States reported an anti-HCV seroprevalence of 20% in adults and 18.5% among children [3]. However a higher prevalence was reported from Egypt 70–80% [4]. HCV infections among patients on haemodialysis were attributed to several risk factors including blood transfusion. A number of studies had revealed a significant correlation between the patients who received blood transfusion and the risk of acquiring HCV infection [5]. The nosocomial risk factors play an important role in HCV infections among patients on haemodialysis, these factors are related to dialysis machines and dialyzers which include dialyzers membranes and haemodialysis ultrafiltrate, reprocessing of dialyzers, and dialysis machines [6]. Haemodialysis staff was found also to be an important factor in transmission of HCV infections among patients on haemodialysis [7]. In order to control the diffusion of HCV in haemodialys units some authors recommended using a separate section to dialyze HCV+ patients [8].

## 2. Patients and Methods

A total of 83 HCV seronegative end stage renal disease (ESRD) patients; who were all the seronegative patients receiving regular haemodialysis during 36 months of the study (2008–2010) in four different haemodialysis units from three different governorates in Egypt were included. A retrospective comparative cohort for two groups of haemodialysis patients was done. **Group 1** (*N* = 27, 18 males, 9 females, mean age 47.14 ± 14.8 yeras, and mean duration on dialysis 70.1 ± 28.9 months) included patients under regular haemodialysis in unit A and unit B, both units are following strict isolation program for HCV seropositive patients by using dedicated areas, machines, and dedicated health-care workers; 12 patients from (**unit A**), Suez hospital (Health insurance organization), Suez governorate, 15 patients from (**unit B**),Vacsera haemodialysis unit, Giza governorate. **Group 2** (*N* = 56, 29 males, 27 females, mean age 50.7 ± 14.0 years, and mean duration on dialysis 60.4 ± 22.01 months) included patients under regular haemodialysis in unit C and unit D, both units are not following strict isolation program for HCV seropositive patients, 21 patients from (**unit C**), New Qalioub hospital, Qalioubiah governorate, and 35 patients from (**unit D**), El-Hamdiah El-Shazliah haemodialysis unit, Giza governorate. During the survey period, all subjects received treatment three times a week. The exclusion criteria included those who did not complete the period of study in their units; either due to death, leaving to other hemodialysis units, or after kidney transplantation. Also patients that had dialysis in multiple units were excluded. Hemodialysis has been performed with cuprophane or polysulphone dialyzers. No dialyzers reuse was performed. The infection control measures recommended were adopted in all units. Hemodialysis machines and environmental surfaces were disinfected after each session. Monthly screening for anti-HCV of all patients. And in addition, anti-HCV positive individuals in units **A** and **B** have been dialyzed on separate dedicated machines. A standardized form was used to collect data on age, sex, history of hepatitis/jaundice, length of time on hemodialysis treatment, number of previous transfusions, tattooing, intravenous drug use, and household contact with hepatitis/jaundice. Permission for carrying out the study was granted by the institutions involved.

### 2.1. Serological Tests

Blood samples were collected from all patients and sera were separated and tested for HCV antibodies using ELISA technique third generation. In this test, diluted patient specimens and controls were incubated in microwells coated with recombinant polypeptides of the structural and nonstructural regions of HCV. If HCV antibodies are present in a specimen or control, they bind to the antigen coated microwell. Excess sample was removed by a wash step and the enzyme tracer then added to the microwells and was allowed to incubate. The enzyme tracer binds to any antigen-antibody complexes present in the microwells. Excess enzyme tracer then removed by a wash step, and a chromogen/substrate solution was added to the microwells and was allowed to incubate. If a sample contains HCV antibodies, the sample turns to a blue colour (650 nm). The blue colour turns to yellow (450 nm) after addition of the stop solution. If a sample does not contain HCV antibodies, the microwell will be colourless. Testing for levels of transaminases (ALT, AST) was done for all the patients including the seroconverted patients.

### 2.2. Statistical Analysis

Data were entered into an I.B.M compatible computer using the statistical package SPSS ver.15. The data were summarized and presented using suitable parameters. Prevalence, incidence, odds ratios, *P*values, and 95% confidence intervals (CI) were calculated to assess differences between studied groups and detect possible risk factors among studied populations. Statistical significance was assessed at 0.05 probability level in all analysis.

## 3. Results

**Figure 1:**
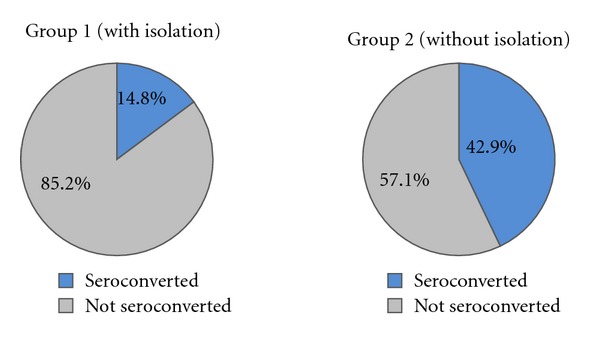
Incidences of seroconversion in both groups.

As shown in Table 1 and Figure 1 there was a significantly higher incidence of HCV seroconversion in patients receiving haemodialysis in units not following strict isolation program for HCV seropositive patients (24 out of 56 patients; 42.9%) than those receiving haemodialysis in units following strict isolation program for HCV seropositive patients (4 out of 27 patients; 14.8%) (*P*-value < 0.05).

Table 2 shows that there was no significant results between seroconverted and not seroconverted patients as regards their age, sex, occupation, marital status, and their education level.

Analysis of risk factors in Table 3 showed that isolation of HCV seropositive patients was associated with a significantly low relative risk for HCV antibody seroconversion (Odds ratio 0.23, *P* value < 0.05), while blood transfusion and duration on regular hemodialysis more than 60 months both were associated with a significant high relative risk for HCV antibody seroconversion (Odds ratio 4.05 and *P* value < 0.05) and (Odds ratio 2.39 and *P* value < 0.05), respectively.

As shown in Table 4 the multivariate “regression” analysis of predictors for seroconversion revealed that the most effective predictors in HCVAb seroconversion were the duration on regular hemodialysis and the isolation of HCV seropositive.

## 4. Discussion

HCV infection still remains a major problem among patients on maintenance HD. The immune suppression seen in this patient population, resulting in an absence of clinical and biochemical evidence of liver disease, is believed to accelerate further dissemination of the virus [9].

The importance of prevention of HCV infection and control is due to its well-documented progression to hepatic cirrhosis, liver malignancies, and liver failure [10].

The prevalence of HCV infection varies greatly among patients on HD from different geographic regions. In a review of data published in 1999, Wreghitt described a range from 4% in the UK to 71% in Kuwait for HCV prevalence among the HD population [11]. In Egypt, the prevalence of HCV antibodies in hemodialysis patients was found to be ranging from 52.3 to 82.3% [12]. Several studies have reported nosocomial patient-to patient transmission of HCV infection among HD patients [13, 14]. 

As a result, in 2001 the *CDC* recommends that special precautions should be observed in dialysis units, including wearing and changing of gloves and waterproof gowns between patients, systematic decontamination of the equipment circuit and surfaces after each patient treatment, and no sharing of instruments (e.g., tourniquets, stethoscope, blood pressure cuff) or medications (e.g., multiuse vials of heparin) among patients. 

Although some studies found that nosocomial spread of HCV declined when HCV-infected patients were treated in dedicated HD units [15, 16], other investigators could control nosocomial spread of HCV by strict application of hygienic precautions without isolation of HCV-infected subjects or machine segregation [17, 18].

In our study we found that the incidence of HCV seroconversion is significantly lower in the group of patients within units implementing isolation programs of the HCV-infected patients than those who had no isolation of the HCV-infected patients.

The duration on regular hemodialysis was found to be a significant predictor for HCV seroconversion in HD patients; a result that is consistent with that in a study in Brazil demonstrated that patients on HD for more than three years had a 13.6-fold greater risk of HCV-positivity compared to subjects with less than one year HD treatment [19]. 

Albeit the use of erythropoietin from the late 1980s reduced the need for blood transfusions among HD patients. Furthermore, the current risk of transfusion-associated HCV is approximately one in every two million people as reported by O'Brien et al., in 2007; mainly after the introduction of nucleic acid amplification testing for the screening of blood donors has markedly reduced the risk of HCV transmission through blood product transfusion [20], however blood transfusion was found in our study to have still a significant relative risk for HCV seroconversion in HD patients (*Odd's ratio* 4.05).

## 5. Conclusions

In HD units with a high prevalence of HCV infection, strict isolation of HCV+ patients in combination with implementation of universal prevention measures are recommended to avoid burden of virus transmission and morbidity.

## Figures and Tables

**Table 1:** Incidence of seroconversion in both groups.

	Group 1		Group 2		*P* value
	No	%	No	%	
Not Seroconverted	23	85.2%	32	57.1%	<0.05
Seroconverted	4	14.8%	24	42.9%	

**Table 2:** Comparison between demographic data of seroconverted and seroconverted patients.

	Total	Not seroconverted	Seroconverted	Incidence of seroconversion	*P* value
Sex					
Male	29	15	14	48.2%	>0.05
Female	27	17	10	37.1%	
Occupation					
No	36	24	12	33.3%	>0.05
Yes	20	8	12	60.0%	
Marital status					
No	6	4	2	33.3%	
Yes	46	25	21	45.6%	>0.05
Widow	4	3	1	25.0%	
Education					
Not	16	10	6	37.5%	
Primary	22	11	11	50.0%	>0.05
Secondary	15	11	4	26.7%	
University	3	0	3	100%	
Age (years)		49.47 ± 15.5	52.29 ± 12.1		>0.05

**Table 3:** Comparison between not seroconverted and seroconverted patients according to risk factors.

	Not seroconverted		Seroconverted		*P* value	Odd's ratio
	No	%	No	%		
Isolation						
With isolation (group 1)	23	41.8%	4	14.3%	<0.05	0.23
Without isolation (group 2)	32	58.2%	24	85.7%		
Duration of hemodialysis						
	21	56.8%	16	43.2%	<0.05	2.39
	35	76.1%	11	23.9%		
Hospitalization						
No	13	23.6%	4	14.3%	>0.05	1.86
Yes	42	76.4%	24	85.7%		
Blood transfusion						
No	18	32.7%	3	10.7%	<0.05	4.05
Yes	37	67.3%	25	89.3%		
Surgery						
No	18	0.0%	11	3.6%	>0.05	1.03
Yes	36	100.0%	17	96.4%		
Tattooing						
No	51	92.7%	26	92.9%	>0.05	0.98
Yes	4	7.3%	2	7.1%		
IV drug abuser						
No	55	100.0%	25	89.3%	<0.05	1.1
Yes	0.0	0.0%	3	10.7%		

**Table 4:** Multivariate “regression” analysis of predictors for HCVAb seroconversion.

	Beta	*T*	Sig.
Duration on hemodialysis	0.433	4.57	0.001
Isolation	−0.267	−2.301	0.01
Hospitalization	0.119	1.037	0.303
Blood Trans.	0.082	0.692	0.426
Surgery	0.223	2.035	0.06
Tattooing	0.018	0.164	0.572
I.V. drug users	0.216	2.016	0.069
